# Not young but still immature: a HIF-1α–mediated maturation checkpoint in regenerating muscle

**DOI:** 10.1172/JCI165322

**Published:** 2022-12-01

**Authors:** Rahagir Salekeen, Michael Kyba

**Affiliations:** 1 Lillehei Heart Institute,; 2 Biochemistry, Molecular Biology, and Biophysics Graduate Program and; 3 Department of Pediatrics, University of Minnesota, Minneapolis, Minnesota, USA.

## Abstract

Muscle fibers express particular isoforms of contractile proteins, depending on the fiber’s function and the organism’s developmental stage. In the adult, after a muscle injury, newly generated fibers transition through embryonic and neonatal myosins, prior to selecting their distinctive adult myosin isoform. In this issue of the *JCI*, Wang et al. discover a checkpoint that regulates the neonatal-to-adult myosin isoform transition. They found that HIF-1α regulated this checkpoint, with elevated HIF-1α levels blocking progression, while HIF-1α knockout accelerated the transition. They further related these findings to centronuclear myopathy, a disease in which HIF-1α is similarly elevated and neonatal myosin expression is maintained. These findings highlight a maturation checkpoint that impacts the skeletal muscle regeneration following ischemic injury, providing a pharmacologically accessible pathway in injury and diseases such as centronuclear myopathy.

## Regeneration parallels embryogenesis

**Figure 1:**
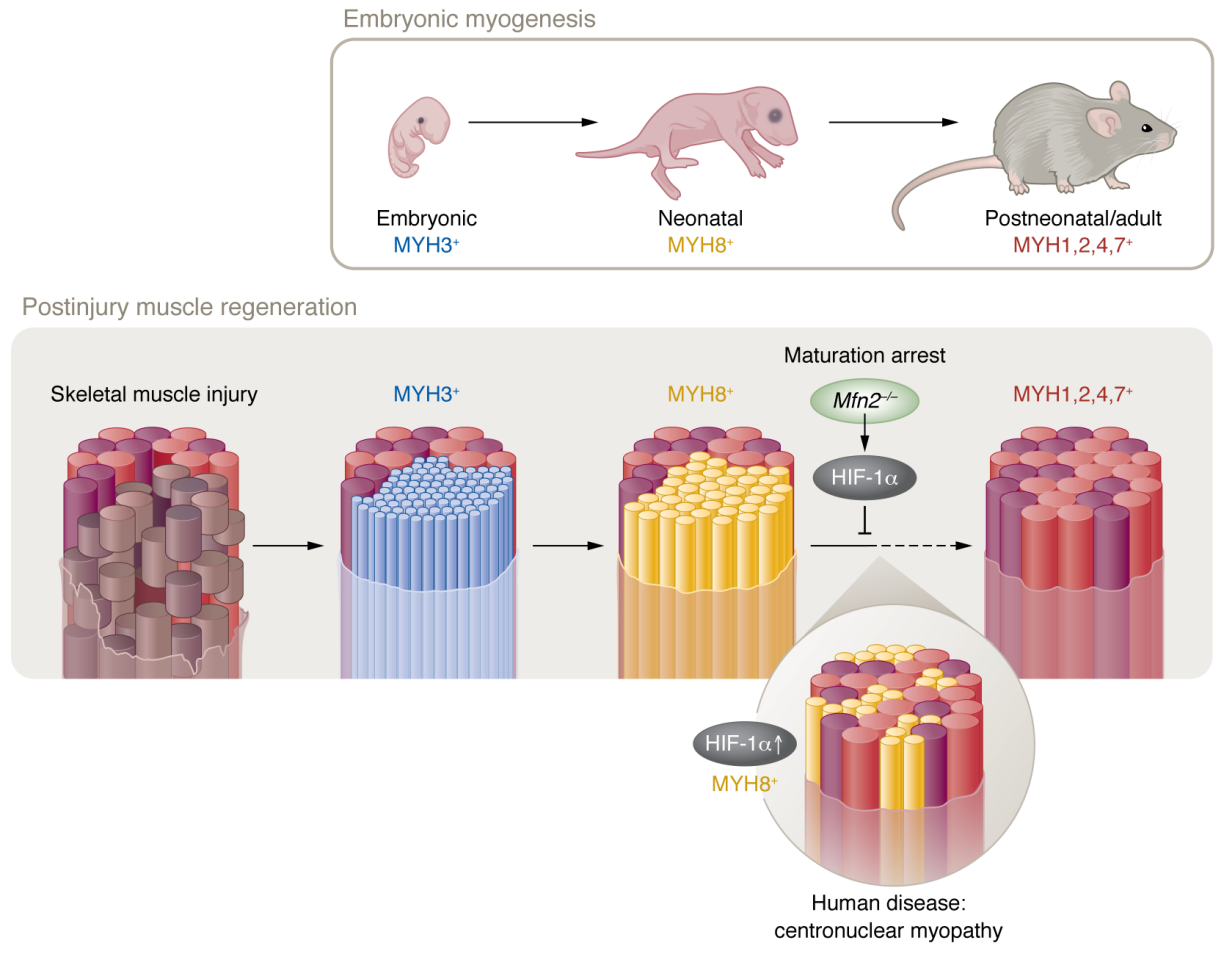
HIF-1α serves as a maturation checkpoint during muscle regeneration after injury. Following injury, newly formed fibers mirror embryonic muscle development, initially expressing embryonic myosin heavy chain (MYH3, blue), and then switching to neonatal myosin (MYH8, yellow) before finally expressing definitive adult myosins appropriate to their particular fiber type (red and purple). MFN2-depleted animals exhibit a maturation arrest phenotype at the neonatal myosin (MYH8^+^) stage. This arrest resembles the pathological phenotype in centronuclear myopathy (shown below). In both cases, prolonged fetal myosin expression is associated with sustained expression of HIF-1α, which serves as a maturation checkpoint.

Skeletal muscle fibers come in many varieties with different properties of metabolism, contractile speed, and fatigability, depending on the physiological role of the muscle they belong to. A total of 11 myosin heavy chains (MyHCs) are expressed throughout the body’s musculature from embryogenesis through adulthood (1). During development, a series of coordinated and sequential myosin type transitions occur wherein the first embryonic fibers, which express myosin heavy chain 3 (MYH3^+^), transition to neonatal MYH8^+^ fibers during late embryonic development. Adult fast-type MYH1/2/4^+^ and adult slow-type MYH7^+^ fibers gradually supplant these immature fibers (1, 2). Interestingly, when muscle is injured and new fibers form, the same expression progression occurs: MYH3^+^ to MYH8^+^, with subsequent adult fast and adult slow MyHC expression (2) (Figure 1).

Different MyHCs are tailored to specific contractile and developmental functions (3), although the isotype-specific molecular roles are yet to be fully elucidated. It has been suggested that prenatal MyHCs may be optimized for lower load bearing, appropriate to the fetal and neonatal muscular demands (1). Germline *Myh3*-KO mice display reductions in fiber size at P0 together with a sharp increase in MYH1/2/8^+^ fibers. These data suggest expression of later isoforms may compensate for absence of earlier isoforms and indicate that distinctions particular to early types of MyHC may not be essential for survival. Costs associated with the precocious expression of adult myosins and possible changes to muscle homeostasis and development remain unknown.

Another parallel between embryonic and regenerative fibers is the low oxygen tension environment that arises from indirect placental blood oxygenation during development or from damage to microvasculature following injury. Hypoxia-inducible factors (HIFs), primarily HIF-1α, have greater activity in low oxygen environments, with HIF-1α retaining stemness of myogenic progenitors and delaying differentiation, providing transient oxygen-tension-responsive energetic conservation, which is reverted with returned oxygen flow after microvasculature reformation/repair and marked by oxidative metabolism (4–6). While a large body of literature on HIF-1α and hypoxic/oxidative stress response in early MYH3^+^ stages of fiber regeneration exists, the later stages of MYH8^+^ to adult-MYH transition dynamics have not been well studied.

## MyoD targets mitochondrial proteins in activated satellite cells

Wang et al. began by comparing MyoD targets in activated cells with quiescent satellite cells, and found that MyoD bound and induced PGC-1β, which in turn induced expression of the mitochondrial protein, mitofusin 2 (MFN2) (7). MFN2 regulates the architecture of the mitochondrial network and has functions in cardiomyocyte aging and maintenance (8–10). Using a conditional satellite cell Cre to delete *Mfn2*, the authors found no notable change in sequential expression of MYOD1, MYOG, and MYH3 up to 5 days post-injury (dpi); however, after 14 dpi, a substantial decrease in muscle and fiber size was observed, together with prolonged retention of MYH8. They also found upregulated methylation modifiers and associated loss of repressive H3K27me3 marks at the *Myh8* locus, correlated with, and possibly catalyzed by, histone lysine demethylases (KDM4 and KDM6), corroborating the expression results, and providing a possible mechanistic intermediate. The lack of transition out of the MYH8^+^ state demonstrates the existence of a maturation checkpoint, and its dependence on MFN2.

## HIF-1α–mediated maturation arrest in *Mfn2*-KO fibers

To better understand the maturation checkpoint, transcriptional profiling of *Mfn2^–/–^* fibers was employed, revealing a strong signal for HIF-1α and its target genes. After injury in WT muscle, HIF-1α expression was high throughout 2 dpi, and then diminished toward 5 dpi, while the KO mice had sustained expression of HIF-1α until 14 dpi. To determine whether HIF-1α levels specified this maturation checkpoint, the authors tested the ability of HIF-1α removal to suppress the *Mfn2^–/–^* 00phenotype. Both chemical suppression (via PX-478, a small molecule inhibitor) and conditional deletion of *Hif1α* in 14 dpi *Mfn2^–/–^* MYH8^+^ fibers resulted in muscle fibers transitioning to the adult-MYH fiber type at 28 dpi, accompanied by increased fiber size and muscle growth.

Having shown that *Hif1α* deletion releases the checkpoint in the context of stalled maturation induced by *Mfn1* mutation, the authors tested the effect of *Hif1α* deletion in regenerating *Mfn2*-WT muscle. After an ischemic injury, MYH8^+^ fibers differentiated into adult fibers substantially earlier in the *Hif1α*-KO compared with WT. Conversely, in the context of sustained HIF-1α expression, decreased fiber sizes and MYH8^+^ stage arrest were observed, together with H3K27me3 deposition at the *Myh8* locus — reminiscent of regeneration in the *Mfn2^–/–^* mice. These data demonstrate conclusively that HIF-1α is an essential component of the MYH8-to–adult MYH maturation checkpoint.

## Clinical implications

Because the checkpoint could be genetically or pharmacologically bypassed by functional removal or suppression of HIF-1α expression, it could have therapeutic potential in diseases in which this checkpoint is affected, such as Charcot-Marie-Tooth disease type 2A, caused by the *MFN2^T105M^* mutation (11, 12). Although the paucity of biopsy samples prevented evaluation of MYH8 in human specimens of this disease, the authors showed that a synonymous mutation in mice led to a MYH8^+^ checkpoint-stalled phenotype similar to that of the *Mfn2*-KO mice. The authors did, however, find such a phenotype in biopsy specimens of centronuclear myopathy (CNM), a disease that also includes abundant small immature fibers; immunostaining revealed large numbers of MYH8^+^ fibers as well as HIF-1α target gene expression. It will be interesting to see whether transient HIF-1α suppression can release the checkpoint in patients with CNM, as well as other diseases with similar fiber phenotypes such as infantile X-linked myotubular myopathy (13), and whether the transition to adult myosin expression would provide any functional improvement for these individuals.

## Connecting the dots

The maturation checkpoint provides context for previous studies of regeneration in the absence of HIF-1α. Larger and denser myofibers have been observed in muscle regenerating after femoral artery ligation in the *Hif1α*-KO mice and although MYH isoforms were not evaluated, Wnt signaling was found to be upregulated, and specific inhibition of Wnt reversed the benefits of the *Hif1α* KO on muscle fiber regeneration (4). Other studies on HIF-1α, although not evaluating MYH8, are consistent with these findings (14, 15). It would therefore be interesting to determine the effect of Wnt inhibition on the *Mfn2^–/–^* or CMT phenotypes.

Also unsettled is the purpose of the maturation checkpoint, or the corollary question: why should the first fibers formed during regeneration transiently have different MyHC isoforms? Load and speed demands on muscle during regeneration are typically limited, meaning embryonic isoforms are sufficient during this time, but the benefit they provide is not really understood. It is interesting that HIF-1α expression in the context of injury is intrinsically self-limiting, with activation of downstream factors such as EPO, VEGF, and GLUT4 promoting microvasculature regeneration, and that glycolytic metabolism is appropriate to the proliferation needs of myogenic progenitors in the early phase of regeneration (5, 14, 16). Germline and/or prolonged HIF-1α/HIF-2α inhibition is reported to decrease the numbers of satellite cells and proliferating myoblasts, and increase their apoptosis (5, 14, 15). Inhibiting HIF-1α and bypassing the checkpoint precociously may therefore be deleterious in some contexts.

The link between mitochondrial dynamics and myofiber maturation arrest revealed by *Mfn2* KO is also interesting. Upregulated HIF-1α expression in mouse cardiac cells has been associated with reduced ischemic injury, less ROS, and inhibition of mitochondrial permeability transition pore (MPTP) opening (9, 10). On the other hand, MFN2 overexpression promotes apoptosis in cardiomyocytes subject to oxidative stress, while inhibition of MFN2 inhibits MPTP formation (8). While not directly related to MYH isoforms, one may hypothesize that a HIF-1α–mediated checkpoint could protect cells from the effects of mitochondrial architecture disruption, facilitating cell survival. How mitochondrial involvement relates to potential benefits of MYH8 expression are dots that remain to be connected.

There are enormous variations in MYH isoform content between muscles, depending on slow- and fast-twitch-type fiber abundance (1, 2, 17, 18). The Wang et al. study focuses on the tibialis anterior, which has abundant fast-twitch fibers, substantial glycolytic metabolism, and greater HIF-1α expression compared with slow oxidative muscles (7). Conditional stabilization of HIF-1α can result in a slow-to-fast fiber type transition in soleus and gastrocnemius muscles (6). Therefore, whether the proposed checkpoint model functions in the same way in different muscle types needs to be investigated. Nevertheless, the discovery of a pharmacologically accessible maturation checkpoint that impacts the rate of skeletal muscle regeneration following ischemic injury, and which appears to be inappropriately active in certain diseases, represents an exciting opportunity for clinical applications.

## Version 1 12/01/2022

Electronic publication
